# Restored Circulating Invariant NKT Cells Are Associated with Viral Control in Patients with Chronic Hepatitis B

**DOI:** 10.1371/journal.pone.0028871

**Published:** 2011-12-16

**Authors:** Xiaotao Jiang, Mingxia Zhang, Qintao Lai, Xuan Huang, Yongyin Li, Jian Sun, William G.H. Abbott, Shiwu Ma, Jinlin Hou

**Affiliations:** 1 Institute of Hepatology and Key Lab for Organ Failure Research, Nanfang Hospital, Southern Medical University, Guangzhou, Guangdong, People's Republic of China; 2 Department of Immunology, Basic Medicine School, Southern Medical University, Guangzhou, Guangdong, People's Republic of China; 3 The New Zealand Liver Transplant Unit, Auckland City Hospital, Auckland, New Zealand; Karolinska Institutet, Sweden

## Abstract

Invariant NKT (iNKT) cells are involved in the pathogenesis of various infectious diseases. However, their role in hepatitis B virus (HBV) infection is not fully understood, especially in human species. In this study, 35 chronic hepatitis B (CHB) patients, 25 inactive carriers (IC) and 36 healthy controls (HC) were enrolled and the proportions of circulating iNKT cells in fresh isolated peripheral blood mononuclear cells (PBMC) were detected by flow cytometry. A longitudinal analysis was also conducted in 19 CHB patients who received antiviral therapy with telbivudine. Thereafter, the immune functions of iNKT cells were evaluated by cytokine secretion and a two-chamber technique. The median frequency of circulating iNKT cells in CHB patients (0.13%) was lower than that in HC (0.24%, P = 0.01) and IC (0.19%, P = 0.02), and increased significantly during antiviral therapy with telbivudine (P = 0.0176). The expressions of CC chemokine receptor 5 (CCR5) and CCR6 were dramatically higher on iNKT cells (82.83%±9.87%, 67.67%±16.83% respectively) than on conventional T cells (30.5%±5.65%, 14.02%±5.92%, both P<0.001) in CHB patients. Furthermore, iNKT cells could migrate toward the CC chemokine ligand 5. Patients with a high ratio (≥1.0) of CD4−/CD4+ iNKT cells at baseline had a higher rate (58.33%) of HBeAg seroconversion than those with a low ratio (<1.0, 0%, P = 0.0174). In conclusion, there is a low frequency of peripheral iNKT cells in CHB patients, which increases to normal levels with viral control. The ratio of CD4−/CD4+ iNKT cells at baseline may be a useful predictor for HBeAg seroconversion in CHB patients on telbivudine therapy.

## Introduction

Worldwide, more than 300 million people suffer from chronic hepatitis B virus (HBV) infection, leading to a wide spectrum of liver diseases including chronic hepatitis B (CHB), cirrhosis, and hepatocellular carcinoma [1]. The pathogenesis of CHB and cirrhosis is thought to be mediated by the immune response to the HBV rather than the HBV itself [2]. Multiple types of immune cells and molecules are involved in HBV associated liver damage. However the precise roles of these cells and molecules are still incompletely understood.

Invariant NKT (iNKT) cells are a unique group of T lymphocytes that express an identical T cell antigen receptor (TCR) α chain, Vα14-Jα18 in mice and Vα24-Jα18 in humans [3]. iNKT cells differ from conventional T lymphocytes in that they recognize lipid or glycolipid antigens presented by the MHC class I-like molecule CD1d. When activated with CD1d tetramer or anti-CD3, iNKT cells rapidly secrete a variety of Th1 and Th2 cytokines within a few hours [4]. Although iNKT cells comprise a very small proportion of peripheral T cells, about 1% in mice and 0.2% in humans, they seem to play important roles in regulating a number of immune responses, including transplant rejection, cancer, autoimmunity, allergy, and infection [5], [6].

The liver contains a larger number of iNKT cells relative to blood and other lymphoid organs [7], [8], [9]. Increasing evidence suggests that iNKT cells contribute to a variety of liver disorders, including drug-induced liver injury [10], [11], primary biliary cirrhosis [12], [13], alcoholic liver injury [14], autoimmune hepatitis [15], hepatocellular carcinoma [16], non-alcoholic fatty liver disease [17], and viral hepatitis [18], [19]. In CHB, alpha-galactosylceramide (α-GalCer) activated iNKT cells are able to inhibit HBV replication in vivo [20] and are implicated in the pathogenesis of cirrhosis by producing profibrotic cytokines [21]. Activation of iNKT cells also promotes the loss of tolerance to HBV-specific CD8+ T cell antigens [22]. However, most of these reports are based on mouse models, and the role of iNKT cells in CHB patients is largely unknown. There are few reports about the changes in iNKT cell frequency or activity in CHB patients during antiviral therapy.

In the present study, we compared the frequency of circulating iNKT cells in 35 CHB patients, 25 inactive HBV carriers and 36 healthy individuals by flow cytometry. We also compared the expression of chemokine receptors on iNKT cells, the ability of iNKT cells to migrate toward different chemokines and the ability to secret cytokines between CHB patients and healthy controls. Finally, we analyzed the longitudinal changes of iNKT cells frequency in CHB patients who received antiviral therapy with telbivudine.

## Methods

### Ethics statement

The study protocol was conducted within the guidelines of the 1975 Declaration of Helsinki, and was approved by the ethics committee of Nanfang Hospital. Written informed consent was obtained from all subjects.

### Patients

Thirty-five chronic hepatitis B (CHB) patients, 25 inactive HBV carriers (IC) and 36 healthy controls (HC) were enrolled in the present study. CHB patients and IC were diagnosed according to the described criteria [23]. The basic clinical characteristics of all subjects were listed in Table 1.The subjects with previous antiviral therapy, with co-infection by the HIV, other hepatitis virus, and with diabetes, severe systemic illness, regular alcohol consumption and hepatocellular carcinoma were excluded.

**Table 1:** Clinical characteristics of the subjects enrolled in the study.

	HC	CHB	IC
Cases (n)	36	35	25
Gender (male/female)	17/19	23/12	12/13
Age (years)	23.5 (21–34)	27 (19–38)	36 (22–55)
Log_10_ HBV DNA (copies/ml)	ND	8.64 (6.19–9.9)	<3
ALT (U/L)	<40	173 (43–834)	<40
AST (U/L)	<40	88 (34–345)	<40
HBsAg positive	0	35	25
HBeAg positive	0	35	0
HBeAb positive	0	0	25
HBcAb positive	0	35	25

**Note.** Data were shown as median (range), unless otherwise noted. HBV, hepatitis B virus; ALT, alanine aminotransferase; AST, aspartate transaminase; ND, not determined.

### Serological assays and HBV DNA assays

The presence of HBsAg, HBsAb, HBeAg, HBeAb and HBcAb was determined using commercial AxSYM MEI kits (Abbott Laboratories, North Chicago, IL). The HBV DNA level was quantified by the Roche Diagnostics Cobas Taqman 48 (Meylan, France), which has a detection limit of 300 HBV-DNA copies/ml.

### Flow cytometry analysis

All the fluorochrome-conjugated antibodies with the isotype controls were purchased from BD Biosciences Pharmingen (San Jose, CA, USA) except for PerCP-anti-CD4 from BioLegend (San Diego, CA, USA).

Ten to thirty milliliter (ml) of heparinized blood was collected from all individuals at the time of recruitment. Of the CHB patients, 19 received anti-viral therapy with telbivudine and were followed up for at least 52 weeks, and additional blood samples were obtained at week 12, week 24 and week 52 during the course of therapy. Peripheral blood mononuclear cells (PBMC) were isolated from whole blood by Ficoll density gradient centrifugation using Lymphoprep™ (AXIS-SHILD, Oslo, Norway). One million fresh isolated PBMC were washed with phosphate-buffered saline (PBS) and resuspended in 100 µl FACS buffer (PBS with 0.5% FBS), and then stained with FITC-anti-CD3, APC-anti-CD4 and PE-anti-iNKT (6B11) at room temperature (RT) for 20 min. After that, cells were washed and analyzed using a BD FACS Canto II cytometer (BD Biosciences, CA, USA) with FACSDiva 5.0 (BD Bioscience, San Jose, CA) and FlowJo (Tree Star Inc., Ashland, OR) software. At least 3 ×10^5^ lymphocytes were gated for each sample.

In some cases, the expression of CC chemokine receptor 5 (CCR5) and CCR6 on peripheral iNKT cells and other T cells was also measured by flow cytometry.

### Intracellular cytokine staining (ICS)

Intracellular cytokine production within peripheral iNKT cells was determined by a four-color flow cytometry as described previously with some modifications [24]. Briefly, two million fresh isolated PBMC were suspended in 200 µl RPMI 1640 supplemented with 10% FBS, and stimulated with 200 ng/ml α-galactosylceramide (α-GalCer, Enzo Life Sciences, NY, USA) or in some cases with 50 ng/ml Phorbol 12-myristate 13-acetate (PMA, Sigma-Aldrich, St. Louis, USA) plus 1 µM calcium ionomycin (Sigma-Aldrich) for 1 hour at 37°C. Brefeldin A (BD Biosciences) was added at 5 µg/ml, and incubation was continued for an additional 8 hours for α-GalCer or 3 hours for PMA stimulation. Cells were then harvested and washed with PBS, and stained with cell surface markers by APC-Cy7-anti-CD3, PerCP-anti-CD4 and PE-anti-iNKT for 20 minutes at RT. The stained cells were washed, fixed and permeabilized with Fix&Perm® reagents (Invitrogen, Carlsbad, CA, USA) according to the manufacturer's instructions. After that, the cells were incubated with either Alexa Fluor 488-anti-IFN-γ or FITC-anti- IL-4 for 20 minutes at RT. Cells were then washed and analyzed by flow cytometry. At least 5 ×10^5^ gated lymphocytes were collected for each sample.

### Migration assay

The ability of recombinant CC chemokine ligand 5 (CCL5) and CCL20 to attract iNKT cells was examined using a transwell system with 24-well and 5 µm pore size (Corning, NY, USA). One and half million fresh isolated PBMC from 3 HC subjects were added to the upper chamber, and either 500 ng/ml CCL5 or CCL20 (PeproTech, NJ, USA) were added to the lower chamber. After 10 hours incubation at 37°C, the cells in the lower chamber were harvested and the frequency of iNKT cells was detected by flow cytometry.

### Statistical Analysis

All statistical tests were performed in GraphPad Prism 5 (GraphPad software, CA, USA). Comparisons between different groups were performed using the Mann- Whitney U test. Within-subject data were compared with the repeated measures ANOVA followed by Tukey's or Bonferroni post-tests. The Spearman rank order correlation coefficient was used for correlation analyses. Categorical variables were compared by Fisher's exact test. The area under receiver operating characteristics (ROC) curves were calculated to assess the use of peripheral iNKT cells frequency at baseline to predict HBeAg seroconversion. For all tests, a two-sided P<0.05 was considered significant.

## Results

### Circulating iNKT cells are decreased in CHB patients

**Figure 1:**
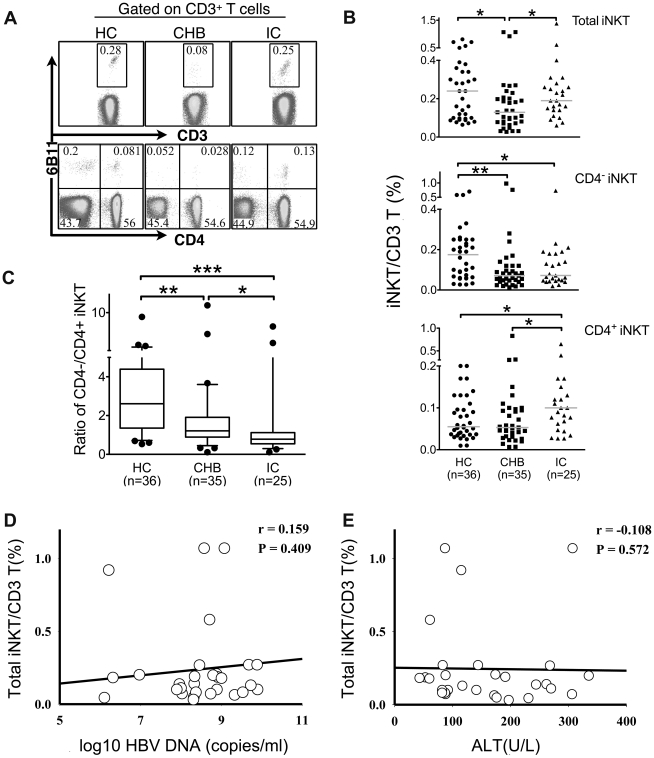
Peripheral invariant NKT (iNKT) cells decreased in CHB patients. Fresh isolated PBMC were stained with FITC-anti-CD3, APC-anti-CD4, and PE-anti-iNKT (6B11), which specifically recognizes the complementarity determining region 3 (CDR3) of the invariant Vα24-Jα18 T cell receptor (TCR) chain, and analyzed by flow cytometry. CD3+6B11+ dot plots indicated iNKT cells, and CD4 was used to determine the subsets of iNKT cells. (A) Representative dot plots of total, CD4− and CD4+ iNKT cells in peripheral blood from healthy controls (HC), CHB patients and inactive carriers (IC). (B) Pooled data of the frequencies of total, CD4− and CD4+ iNKT cells in three groups. Horizontal bars indicated the median value of each group. (C) The ratio of CD4−/CD4+ iNKT in HC, CHB and IC. Horizontal lines illustrated the 25^th^, 50^th^, and 75^th^ percentiles. (D, E) Spearman correlation test showed that the frequencies of total iNKT cells in CHB patients (n = 30) had no correlation with HBV DNA and alanine transaminase (ALT) levels. * P<0.05, ** P<0.01, *** P<0.001, using the Mann-Whitney U test.

We first compared the frequencies of peripheral iNKT cells in subjects from the HC, CHB and IC groups. iNKT cells were detected by flow cytometry using a monoclonal antibody 6B11 (Fig. 1A), which specifically reacts with the complementarity determining region 3 (CDR3) of the Vα24-Jα18 T cell receptor of human iNKT cells [25]. We found that the frequency (medium, range) of iNKT cells in CHB patients (0.13%, 0.027%–1.071%) was significantly lower than that in HC (0.24%, 0.064%–0.81%, P = 0.0143) and IC (0.19%, 0.06%–0.38%, P = 0.0235). By contrast, there was no significant difference between HC and IC (P = 0.703, Fig. 1B).

iNKT cells are heterogeneous and can be categorized into two subsets according to the expression of CD4, namely CD4+ and CD4− iNKT cells [26]. A comparison of the frequency of CD4+ and CD4− iNKT cells was performed in the three groups. We found that the frequency of CD4− iNKT cells in CHB patients (0.071%, 0.013%–0.98%) was decreased compared to HC (0.175%, 0.027%–0.7%, P<0.01), and the frequency of CD4− iNKT cells in IC (0.072%, 0.019%–0.73%) was also significantly lower compared to HC (P = 0.023). There was no significant difference (P = 0.458) between CHB and IC in the frequency of CD4− iNKT cells (Fig. 1B). By contrast, the frequency of CD4+ iNKT cells in CHB patients (0.053%, 0.007%–0.83%) was significantly lower than IC (0.1%, 0.027%–0.65%, P = 0.019), but was similar to HC (0.055%, 0.01%–0.2%, P = 0.7, Fig. 1B).

We next compared the ratio of CD4−/CD4+ iNKT cells in the different groups, and found that the ratio was decreased in CHB patients (1.21, 0.11–10.77) relative to the HC (2.61, 0.53–9.55, P<0.01), and even further decreased in the IC (0.78, 0.11–8.5) relative to CHB (P = 0.018, Fig. 1C). We also analyzed the correlation between peripheral iNKT cells and plasma HBV DNA load and serum alanine aminotransferase (ALT) level in the CHB patients (n = 30). The results indicated that there was no correlation between the frequency of total iNKT cells and plasma HBV DNA load or serum ALT level (Fig. 1D and 1E). By contrast, CD4− iNKT cells and the ratio of CD4−/CD4+ iNKT cells were negatively correlated with ALT levels (Fig. S1).

### iNKT cells express high levels of CCR5 and CCR6

**Figure 2:**
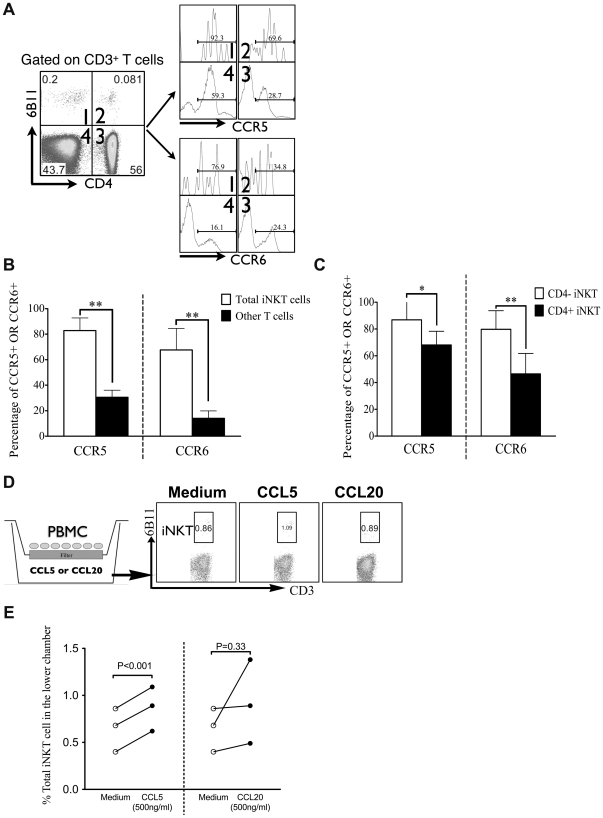
Peripheral iNKT cells expressed high levels of CC chemokine receptor 5 (CCR5) and CCR6. (A) Representative dot plots of CD3+6B11+ iNKT and CD3+6B11- T (Other T) cells from CHB patients (n = 6) and histograms of CCR5 and CCR6 expression within each quadrant. (B, C) Pooled data compared the expression of CCR5 and CCR6 between iNKT and other T cells, between CD4− and CD4+ iNKT cells from CHB patients. *P<0.05, **P<0.01, using Mann-Whitney U test. (D) Schematic diagram of migration assay. Fresh isolated PBMC were added into the upper chamber, 500 ng/ml CC chemokine ligand 5 (CCL5), CCL20 or medium alone was added into the lower chamber. Ten hours later, the cells of the lower chamber were harvested and analyzed. (E) Pooled data compared the percentages of iNKT cells in the lower chamber using paired t-test.

Next, we detected the expression of CCR5 and CCR6 on peripheral iNKT cells in CHB patients (n = 6) by flow cytometry (Fig. 2A) and found that the proportion (mean±SD) of iNKT cells expressing CCR5 (82.83%±9.87%) or CCR6 (67.67%±16.83%) was dramatically higher than that of other T cells (CCR5, 30.5%±5.65%, CCR6, 14.02%±5.92%, both P<0.001, Fig. 2B). Further analysis revealed that the percentage of CD4− iNKT cells expressing CCR5 or CCR6 was also significantly higher than that of CD4+ iNKT cells (P<0.05 and P<0.01 respectively, Fig. 2C). Interestingly, the expression of CCR5 and CCR6 on iNKT cells was comparable between CHB patients and HC subjects (Fig S2).

Our data above indicated that iNKT cells expressed higher levels of CCR5 and CCR6 than other T cells. Therefore, we performed a chemotaxis assay to test the hypothesis that CCL5 and CCL20, the ligands of CCR5 and CCR6 respectively, could induce the migration of iNKT cells in vitro. We found that the frequency of iNKT cells in the lower chamber in response to CCL5 (0.87%±0.24%) was significantly higher compared to medium alone (0.65%±0.23%, P<0.001). Surprisingly, the frequency of iNKT cells in the lower chamber in response to CCL20 was similar to medium alone (Fig. 2D and 2E).

### Cytokine-secreting profile of iNKT cells in CHB patients

**Figure 3:**
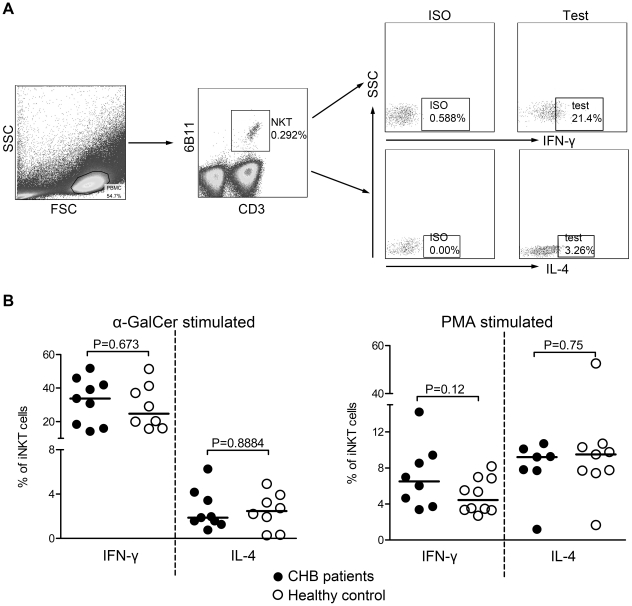
Cytokine secretion of iNKT cells in CHB patients and healthy controls. Fresh isolated PBMC were stimulated with phorbol 12-myristate 13-acetate (PMA) or α-galactosyl ceramide (α-GalCer), a CD1d-restricted antigen recognized specifically by iNKT cells, and the intracellular cytokine were analyzed by flow cytometry. (A) The gating strategy of CD3+6B11+ iNKT cells and representative dot plots of interferon-gamma (IFN-γ) and interleukin-4 (IL-4) expression in iNKT cells from testing samples and isotype controls. (B) Pooled data showed the percentages of iNKT cells producing IFN-γ and IL-4 in response to α-GalCer (left) or PMA (right) in CHB patients and healthy controls. Comparisons between two groups were performed using Mann- Whitney U test.

We also assessed the ability of iNKT cells to produce IFN-γ and IL-4 in response to the specific antigen α-GalCer and non-specific mitogen PMA by flow cytometry. As shown in Fig. 3, the IFN-γ production of circulating iNKT cells in response to α-GalCer was dramatically higher compared to IL-4, irrespective of whether they were obtained from CHB patients or HC (both P<0.001). Interestingly, there were no differences between the two groups in both IFN-γ (P = 0.673) and IL-4 expression (P = 0.8884). When stimulated with PMA, there were also no significant differences between CHB patients and HC in IFN-γ and IL-4 production (P = 0.12, P = 0.75 respectively). Surprisingly, the IFN-γ response to PMA was lower compared with the response to α-GalCer, regardless of CHB patients or HC (both P<0.001). Taken together, these data demonstrate that the ability of peripheral iNKT cells to produce IFN-γ and IL-4 is not impaired with chronic HBV infection and IFN-γ is the dominant cytokine secreted by circulating iNKT cells in response to α-GalCer.

### Decreased iNKT cell frequency recovers after telbivudine therapy

**Figure 4:**
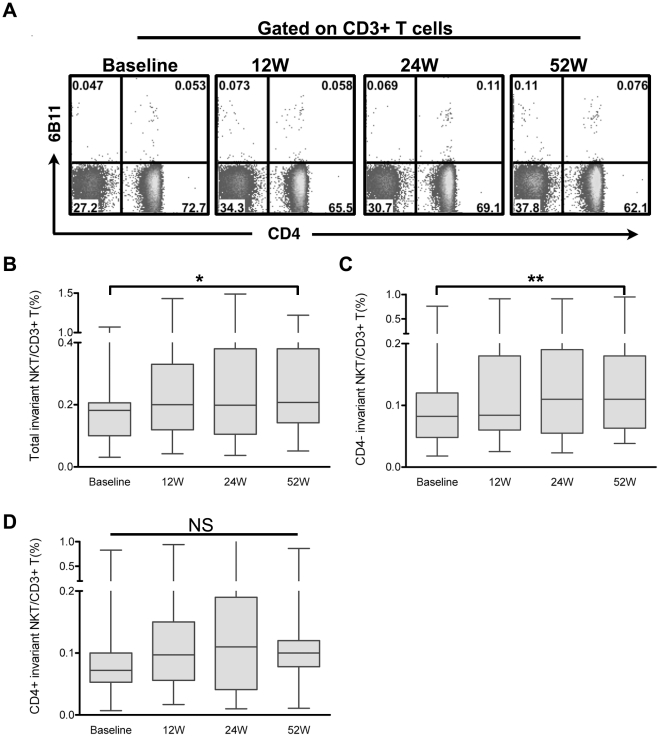
Circulating iNKT cells of CHB patients were recovered during antiviral therapy. (A) Representative dot plots. (B–D) Pooled data showing the dynamic changes of total, CD4− and CD4-iNKT cells at different time points in CHB patients (n = 19) during antiviral therapy. * P<0.05, ** P<0.01, using repeated measures ANOVA followed by Tukey's Multiple Comparison Test; NS, no significance.

We next analyzed the changes in peripheral iNKT cell frequency in CHB patients on antiviral therapy (Fig. 4A). PBMC were obtained at baseline and at week 12, 24 and 52 after initiating treatment with telbivudine (n = 19). We found that the frequency of peripheral iNKT cells was significantly increased at week 52 (0.21%, 0.05%–1.22%) compared with baseline (0.18%, 0.03%–1.07%, P = 0.0176). At week 12 and week 24, the frequency of circulating iNKT cells was also increased compared to the baseline, but the difference did not reach statistical significance (Fig. 4B). Further analysis indicated that the frequency of CD4− but not CD4+ iNKT cells was dramatically increased by week 52 (P<0.01, P = 0.2898 respectively, Fig. 4C and 4D).

### The ratio of CD4−/CD4+ iNKT cells in CHB patients is associated with HBeAg seroconversion

Of the 19 CHB patients treated with telbivudine, 7 (37%) achieved HBeAg seroconversion by week 52. The plasma HBV DNA load in patients with seroconversion decreased more rapidly than patients not achieving seroconversion during the course of therapy, especially after week 12 (Fig S3A). By contrast, both the seroconversion and non-seroconversion patients had the similar variation mode in serum ALT levels and circulating iNKT cells (Fig S3B–F).

**Figure 5:**
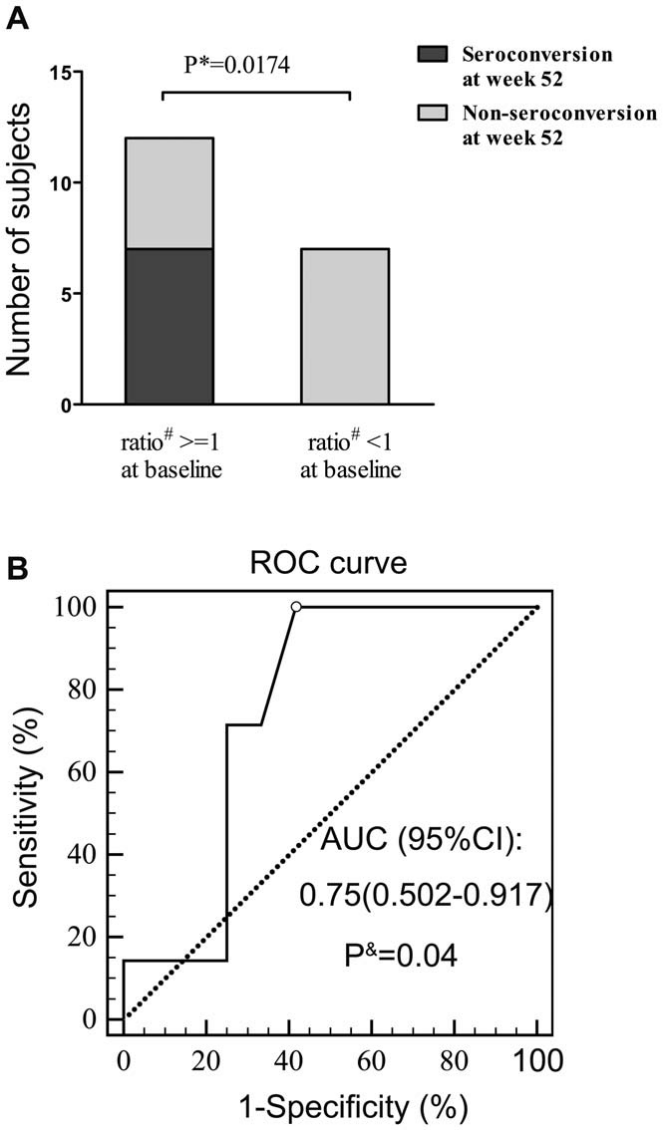
The ratio of CD4−/CD4+ iNKT cells are associated with HBeAg seroconversion. CHB patients who received telbivudine (n = 19) were divided into two groups according to the ratio of CD4−/CD4+ iNKT cells at baseline. (A) The number of patients achieving HBeAg seroconversion in the high (> = 1) and low (<1) ratio groups. (B) Receiver operating characteristics (ROC) curve showing the relationship between CD4−/CD4+ iNKT cells and HBeAg seroconversion. * Fisher's exact test; # The ratio of CD4−/CD4+ iNKT cells; & DeLong test; AUC, Area under ROC curve; 95% CI, 95% confidence interval.

The precise mechanisms contributing to HBeAg seroconversion during antiviral therapy are not fully understood. As showed in Table 2, the patients who achieved HBeAg seroconversion tended to have a slightly higher ratio of CD4−/CD4+ iNKT cells (1.47, 1–3.56) compared to those who failed to achieve HBeAg seroconversion (0.915, 0.11–3.4, P = 0.0826). We also analyzed this data by dividing the patients into high (≥1) and low (<1) ratio groups and found that the patients with a high ratio of CD4−/CD4+ iNKT cells at baseline were more likely to achieve HBeAg serocon- version than those with a low ratio (P = 0.02, Fig. 5A).

**Table 2:** Baseline clinical characteristics of 19 CHB patients who received telbivudine.

	Seroconversion at week 52	Non-seroconversion at week 52	P value
Cases (n)	7	12	
Age (years)	26 (19–38)	27.5 (20–35)	0.6363
Gender (male/female)	4/3	9/3	0.6169
Log_10_ HBV DNA (copies/ml)	8.34(6.97–8.88)	8.77 (6.22–9.73)	0.5314
ALT (IU/L)	191 (87–270)	84.5 (43–335)	0.1837
CD4− iNKT/CD3+ T cells (%)	0.12 (0.041–0.17)	0.076(0.018–0.76)	0.3976
CD4+ iNKT/CD3+ T cells (%)	0.057 (0.04–0.097)	0.094 (0.004–0.83)	0.2906
Total iNKT/CD3+ T cells (%)	0.19 (0.081–0.267)	0.18 (0.027–1.07)	0.7352
Ratio of CD4−/CD4+ iNKT cells	1.47 (1–3.56)	0.915 (0.11–3.4)	0.0826

**Note.** Data are shown as median (range), unless otherwise noted. HBV, hepatitis B virus; ALT, alanine aminotransferase; iNKT, invariant NKT cells. Comparisons of different clinical characteristics between two groups were performed using the Mann-Whitney U test except the gender, which was tested by Fisher's exact test.

Next, a receiver operating characteristics (ROC) curve was constructed to determine whether the ratio of CD4−/CD4+ iNKT cells could be used as a predictor of HBeAg seroconversion within 52 weeks of starting telbivudine therapy. The area under the ROC curve (AUC) indicated the existence of a significant association between the CD4−/CD4+ iNKT ratio and HBeAg seroconversion (AUC = 0.75, 95% confidence interval 0.507–0.993, P = 0.04). A CD4−/CD4+ iNKT cell ratio cutoff at baseline of 0.94 gave the maximum combination of sensitivity (100%) and specificity (58.33%) to predict HBeAg seroconversion (Fig. 5B). Taken together, these data suggest that the ratio of CD4−/CD4+ iNKT cells in CHB patients at baseline is associated with HBeAg seroconversion by week 52 after telbivudine therapy, and has the potential to serve as a predictor for HBeAg seroconversion.

## Discussion

iNKT cells are a heterogeneous immune cell type that may be involved in both the up- and down- regulation of a wide range of immune reactions, including those to the hepatitis B virus [27], [28]. In the context of HBV infection, the majority of iNKT cell researches have been carried out in animals, and the behaviour of these cells in humans with chronic HBV infection is poorly understood. The purpose of this study was to test the hypothesis that iNKT cells influence the clinical status of human subjects with a chronic HBV infection. First, we conducted a cross-sectional study to look for associations between iNKT cell subset frequencies in PBMC and the clinical status of subjects with a chronic HBV infection. Second we tested hypotheses relating to the possibility that chronic hepatitis B is associated with the migration of iNKT cells into the liver. Finally we looked for changes in iNKT cell subset frequencies that were associated with and preceded changes in clinical status in longitudinal data.

We first measured the proportion of circulating iNKT cells cross-sectionally in CHB patients, healthy subjects and inactive HBV healthy carriers. The frequency of circulating iNKT cells was significantly decreased in CHB patients. De Lalla et al. reported recently that the median percentage of peripheral iNKT cells within total T cells was 0.09% in both chronic HBV- and HCV- infected patients without cirrhosis, and 0.2% in healthy donors. Our results are consistent with these findings [21]. In contrast, Inoue et al. found no difference in frequency of iNKT cells between patients with a chronic HCV infection and healthy subjects [29]. These results suggest either that iNKT cells have different roles in different viral infections or that there are unknown differences in the populations of subjects studied.

iNKT cells are phenotypically and functionally heterogeneous. CD4+ iNKT cells are able to produce Th1 and Th2 cytokines and tend to induce tolerance. By contrast, CD4− iNKT cells are more likely to induce a Th1 response [26]. Therefore we analyzed the variation of the two subsets of iNKT cells in CHB patients, and found that the frequency of CD4− iNKT cells was decreased in CHB, whereas the frequency of CD4+ iNKT cells was similar to healthy subjects. As with Th1 and Th2 cells, it is possible that there is a balance between CD4+ and CD4− iNKT cells in healthy subjects, and the balance changes in various clinical diseases, including chronic HBV infection. In the aspect of cytokine-secretion, our data indicated that there was no difference between CHB patients and HC in IFN-γ and IL-4 production of circulating iNKT cells when stimulated with α-GalCer or PMA. Snyder-Cappione JE et al. described recently that the IFN-γ response of circulating iNKT cells to α-GalCer was impaired in HIV infected patients, suggesting the functions of iNKT cells may be distinct among different virus infection [24].

The precise mechanism of the decrease in circulating iNKT cells in CHB patients remains unclear. Based on the report that intrahepatic iNKT cells are dramatically enriched in chronically inflamed livers as compared with noninflamed ones, we suppose that the decrease in CHB patients is at least partly due to trafficking to the liver [21]. The migration of circulating leukocytes to sites of inflammation or injury is tightly directed by chemokine and chemokine receptor interactions [30]. In patients with chronic HCV infection, the expression of CCR1 and CCR5 on peripheral CD8+ T cells was lower and the migration of CD8+ T cells in response to MIP-1α (CCL3), MIP-1β (CCL4), and RANTES (CCL5) was significantly reduced compared with healthy subjects [31]. In chronic HBV infection, there are substantial numbers of virus-specific CD8 T cells, which are barely detectable in the peripheral blood, infiltrated into the liver [32], [33]. It has been reported that cytotoxic T lymphocytes specific for HBV, HCV or HIV-1 could release large quantity of CCL3, CCL4 and CCL5, the ligands of CCR5, when activated with viral antigen expressed on infected cells [34]. Considering the high expression of CCR5 and CCR6 on circulating NKT cells in CHB patients and the migration ability toward CCL5 in vitro in our study, it's reasonable to suppose that iNKT cells may recruit to the liver in CHB patients, leading to the increased proportion of intrahepatic iNKT cells and decreased proportion of peripheral iNKT cells. Whereas in healthy control, there is no inflammation and rare virus-specific CD8 T cells within the liver, resulting in a relatively lower amount of intrahepatic chemokines compared to the CHB patients. Consequently, although the expression of CCR5 and CCR6 on iNKT cells is comparable high with CHB patients, the recruitment of circulating iNKT cells to the liver in healthy control is not as strong as that in CHB patients, causing higher percentage of circulating iNKT cells in HC indicated in our results.

There are very few studies in which changes in iNKT cell frequency have been shown in longitudinal data from CHB patients. The present study shows that the proportion of peripheral iNKT cells in CHB patients was significantly increased at 52 weeks after beginning telbivudine therapy, and approached the levels found in inactive healthy carriers. Interestingly, only CD4− iNKT cells increased at week 52, consisting with the results of cross-sectional study that only CD4− iNKT cells decreased in CHB patients. Thus both data suggest that there is an association between control of the virus, whether as a result of host immunity or antiviral therapy, and higher levels of iNKT cells. These data also support our hypothesis that there is a balance between the frequencies of CD4+ and CD4− iNKT cells, and viral clearance will restore the balance which was altered by the chronic viral infection.

Here, we show that the frequency of peripheral iNKT cells was significantly decreased in patients with CHB relative to both healthy controls and inactive healthy HBV carriers. Moreover, the expressions of CCR5 and CCR6, whose ligands are preferentially expressed in the liver, were significantly higher on iNKT cells than that on conventional T cells. This may partially explain why iNKT cells are enriched in the liver. In addition, the low frequency of iNKT cells in CHB patients increased on antiviral therapy. The ratio of CD4−/CD4+ iNKT cells at baseline was positively associated with HBeAg seroconversion after 52 weeks antiviral therapy with telbivudine, and may be useful as a predictor of HBeAg seroconversion on telbivudine therapy in CHB patients.

## Supporting Information

Figure S1 **Correlations of iNKT cells with either HBV DNA or ALT levels.** The Spearman rank order correlation test was used to evaluate the correlations of CD4+ iNKT cells (A, B), CD4− iNKT cells (C, D) and the ratio of CD4−/CD4+ iNKT cells (E, F) with HBV DNA or ALT levels in CHB patients (n = 30).
(TIF)

Figure S2 **The CCR5 and CCR6 expression on iNKT cells between CHB patients and HC.** The proportion of total iNKT cells expressing CCR5 and CCR6 in CHB patients (n = 6) was compared with HC subjects (n = 10) using Mann-Whitney U test. NS means no significance.
(TIF)

Figure S3 **The dynamic changes of iNKT cells in CHB patients during therapy.** Nineteen CHB patients received anti-viral therapy with telbivudine were divided into two groups, seroconversion (n = 7) and Non-seroconversion (n = 12), depending on the achievement of HBeAg seroconversion at week 52. The dynamic changes of HBV DNA and ALT levels (A, B), total, CD4− and CD4+ iNKT cells (C–E), and the ratio of CD4−/CD4+ iNKT cells (F) at different time points were compared between two groups. *P<0.05, **P<0.01, using repeated measures two-way ANOVA followed by Bonferroni post-tests.
(TIF)
