# Supplementary figures and images for: Restored Circulating Invariant NKT Cells Are Associated with Viral Control in Patients with Chronic Hepatitis B
Source: PLoS One. 2011 Dec 16;6(12):e28871. doi: 10.1371/journal.pone.0028871 (PMC3241692; doi:10.1371/journal.pone.0028871)

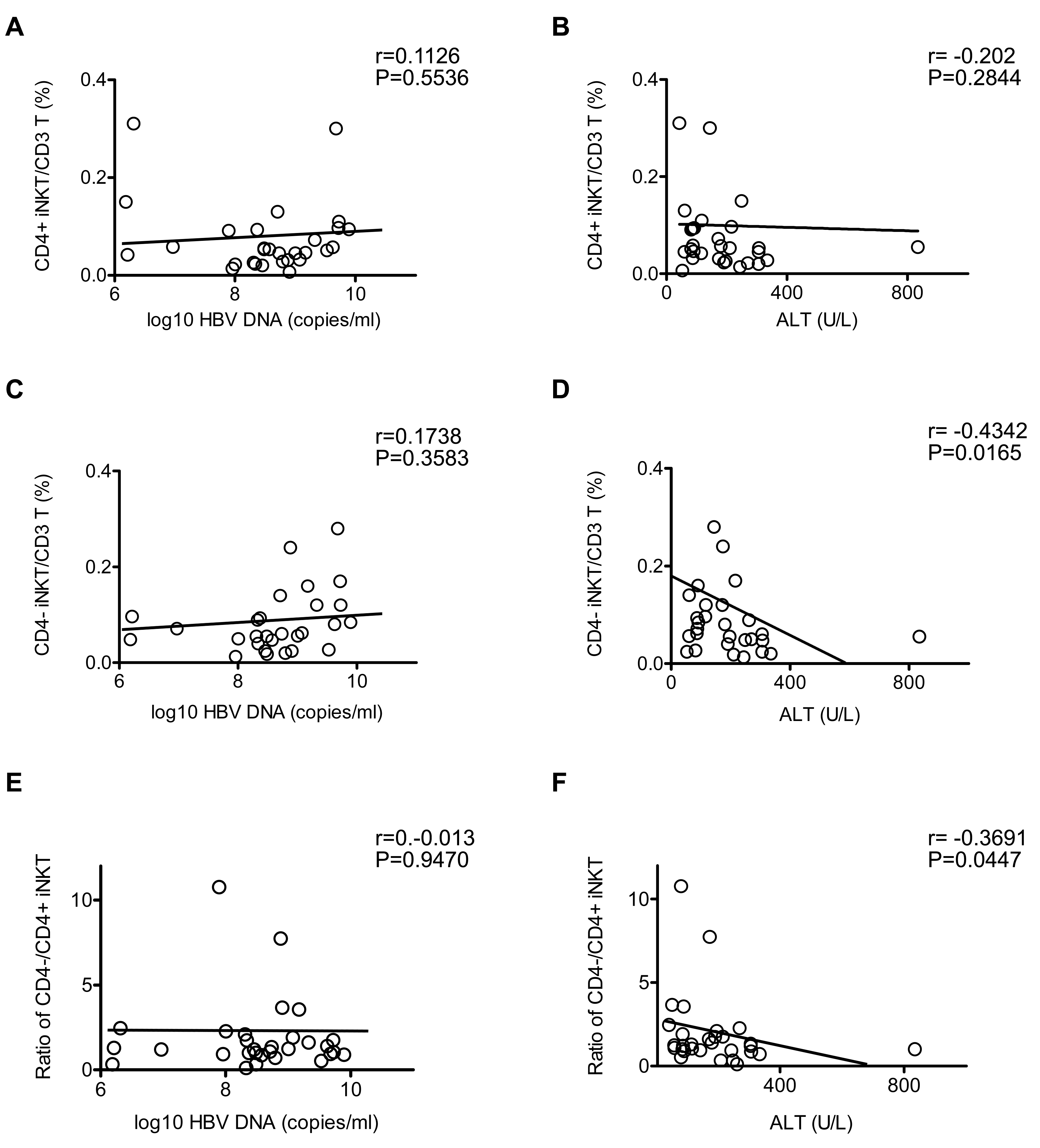

Supplement: Figure S1 — Correlations of iNKT cells with either HBV DNA or ALT levels. The Spearman rank order correlation test was used to evaluate the correlations of CD4+ iNKT cells (A, B), CD4− iNKT cells (C, D) and the ratio of CD4−/CD4+ iNKT cells (E, F) with HBV DNA or ALT levels in CHB patients (n = 30). (TIF) [file pone.0028871.s001.tif]

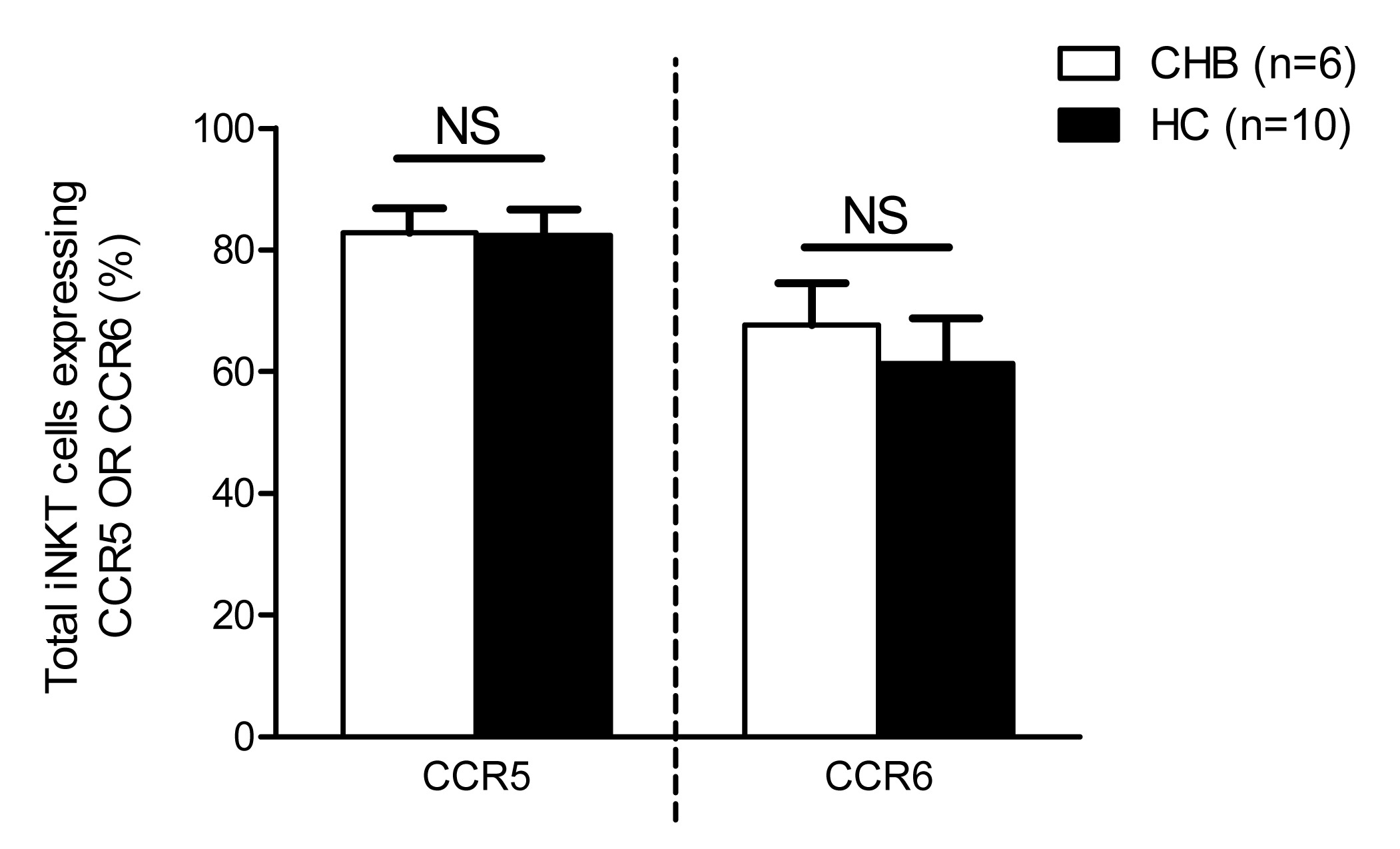

Supplement: Figure S2 — The CCR5 and CCR6 expression on iNKT cells between CHB patients and HC. The proportion of total iNKT cells expressing CCR5 and CCR6 in CHB patients (n = 6) was compared with HC subjects (n = 10) using Mann-Whitney U test. NS means no significance. (TIF) [file pone.0028871.s002.tif]

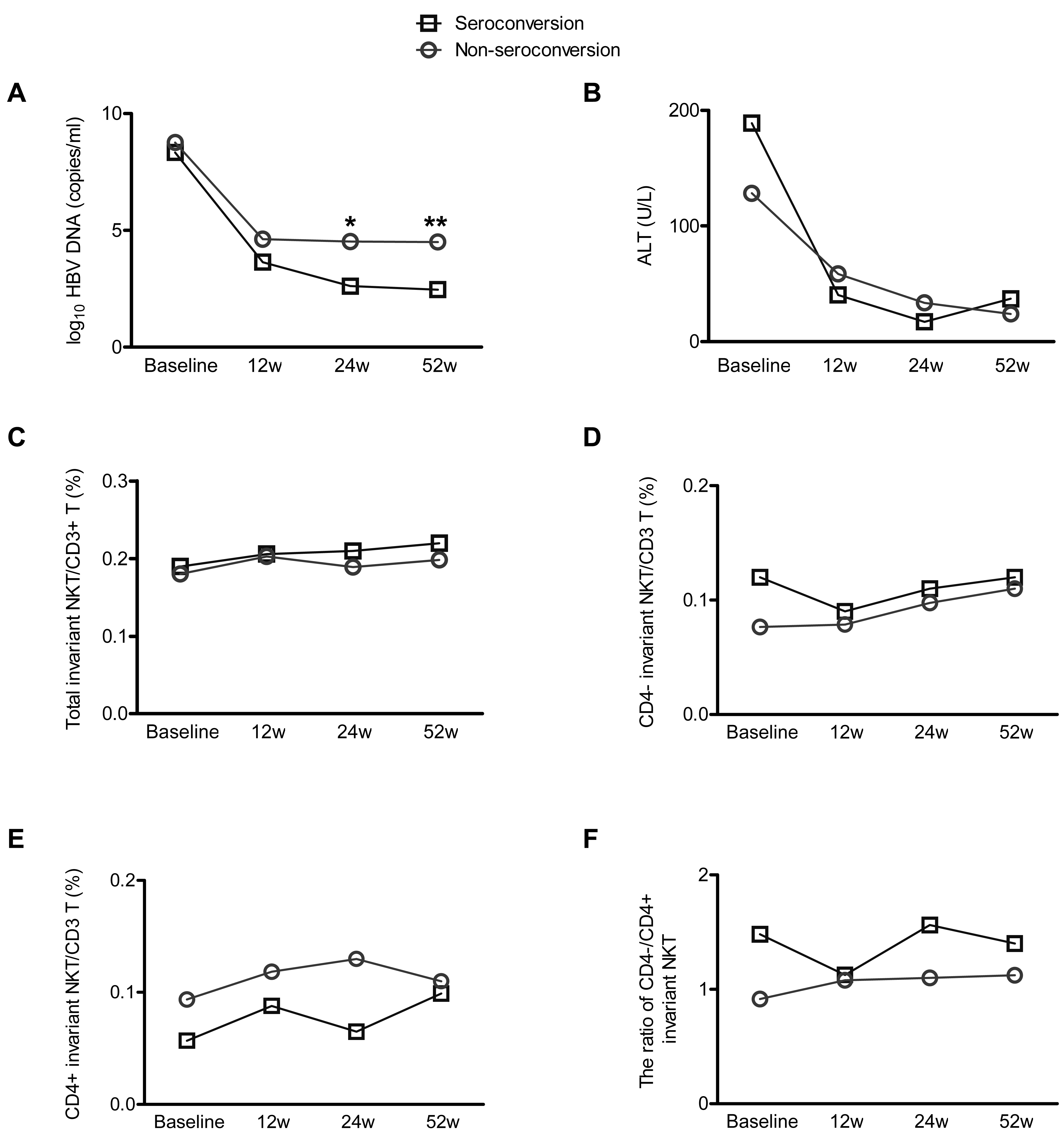

Supplement: Figure S3 — The dynamic changes of iNKT cells in CHB patients during therapy. Nineteen CHB patients received anti-viral therapy with telbivudine were divided into two groups, seroconversion (n = 7) and Non-seroconversion (n = 12), depending on the achievement of HBeAg seroconversion at week 52. The dynamic changes of HBV DNA and ALT levels (A, B), total, CD4− and CD4+ iNKT cells (C–E), and the ratio of CD4−/CD4+ iNKT cells (F) at different time points were compared between two groups. *P<0.05, **P<0.01, using repeated measures two-way ANOVA followed by Bonferroni post-tests. (TIF) [file pone.0028871.s003.tif]
